# ‘Home Is Where You Are Together’: Qualitative Systematic Review and Meta‐Synthesis of Homeless People's Description of Home

**DOI:** 10.1111/hex.70683

**Published:** 2026-05-05

**Authors:** Leila Thornhill, Ben Sellar, Gabrielle Rosa Hernandez, Carolyn M. Murray

**Affiliations:** ^1^ School of Allied Health and Human Performance, IIMPACT in Health Adelaide University Adelaide South Australia Australia

**Keywords:** belonging, homelessness, identity, meaning of home, protection, relationships, social connections

## Abstract

**Background and Objective:**

Homelessness is a pressing worldwide social and health concern. People who are housed may not necessarily adapt to being at ‘home’ and return to homelessness. To learn more, this study explored and synthesised how people with lived experiences of homelessness described the concept of ‘home’. A meta‐ethnographic qualitative systematic review was conducted to provide insights for improving housing outcomes and person‐centred policies and programmes.

**Search Strategy:**

The search strategy was developed in Medline in consultation with an academic librarian and adapted for other databases. Seven databases and grey literature were systematically searched in March 2025. After removing duplicates, 3,013 records were screened in duplicate, with 44 included.

**Inclusion Criteria:**

Qualitative and mixed methods studies published in English were included, focusing on people aged 16–65. Included studies were appraised using a Reporting Assessment Checklist and the Critical Appraisal Skills Programme.

**Data Extraction and Synthesis:**

Participant quotes and interpretations of data were extracted and coded. Data synthesis translated concepts across studies using first‐, second‐ and third‐order analysis.

**Results and Discussion:**

Three themes were developed. Theme one (‘home is stability and return to self’) described a consistent and stable home as the foundation for identity development and outlook. Home was more than a physical space but the foundation for thriving. Theme two described home as a ‘protective and controllable threshold’ allowing the safety and space for reflection, and self‐care which promotes health and well‐being. In theme three (‘home is having connection’), home facilitated temporal, spiritual, personal, and community connectedness; often achieved through engagement in occupations which cultivated inclusion.

**Conclusion:**

Home is more than shelter; it is a place to dream, feel safe, healthy, and part of society. It is recommended that the concepts of stability, identity, connectedness, protection, self‐care, inclusion and occupation be incorporated into improving the transition into housing and for supporting future housing outcomes for homeless people.

**Patient or Public Contribution:**

A consumer representative who was independent of the research team and process was consulted during data synthesis to check the developing themes and discuss their implications and language used. Some changes were made following this consultation.

## 1 Introduction

Homelessness is a pressing social and health concern, with roughly 150 million people experiencing homelessness worldwide [1]. Homelessness has typically been attributed to a shortage of resources to meet housing needs [2, 3]. However, homelessness stems from more than the absence of a residence and having a house does not always resolve underlying unmet needs and structural factors. To appropriately support transitioning to ‘homeness’ [4] it is essential to consider how a physical space becomes a place that feels like home. The challenge of transitioning into a stable home requires consideration of individual factors and needs [5].

Homelessness is typically defined as either primary, secondary or tertiary [6]. Primary homelessness includes those living on the streets or in improvised shelters [6]. Secondary homelessness involves individuals moving between temporary shelters, including emergency accommodations, or couch‐surfing [6]. Tertiary homelessness refers to individuals living in substandard conditions, such as caravan parks or boarding houses that fall below the cultural norm [6]. Recent increases in homelessness have been linked to rising costs of living [7], which have outpaced income growth [7], coupled with a shortage of public housing [2]. This trend is particularly noticeable in high to middle‐income countries [2]. This intensification in determinants of homelessness has not only led to higher rates of homelessness but a greater diversity of the people affected [8]. Compared to the situation 20 years ago, there is now a higher representation of families, women, younger, and older people [8] among those who identify as homeless. Without proactive efforts to increase the efficacy of prevention and intervention, the number of people experiencing homelessness is likely to worsen.

People who are homeless typically need more than just housing support. Many people have disabilities or health issues, including cognitive impairments (30‐40%) [1], mental health issues (80%), and physical health problems (41%) [9]. Certain groups, including people of colour, Indigenous people, and LGBTQIA+ people, are also disproportionately represented among homeless populations [10]. Statistics indicate that risk factors for homelessness include racism, low socioeconomic status, limited education and skills, substance misuse, involvement in the justice system, family/domestic violence, and breakdowns in personal relationships [11]. Moreover, homelessness itself is traumatising, and compounds existing inequities [12], compromising future tenancy [2, 12]. Even when people can secure housing [10, 13], trauma, loneliness, and boredom can continue exacerbating mental and physical health issues and lead to misadventure and involvement in criminal activities [14]. Arguably, homelessness stems from more than just the absence of a residence [15] and having a house does not always resolve the underlying unmet needs and structural factors associated with homelessness [13, 16].

Efforts to tackle homelessness have changed over time. Historically, housing has been tied to behaviour change such as abstaining from drugs and alcohol or engaging in therapies [17, 18, 19]. However, in the past few decades [20], the ‘Housing First’ approach has emerged and become the ‘gold standard’ in high‐income countries [8]. Housing First provides immediate and unconditional access to stable housing as the foundational step in addressing people's needs [8]. At the same time, policy and organisational aspirations have shifted away from the provision of shelter and towards a notion of home. Emerging terms like ‘homing’ [4, 20, 21], and ‘homeness’ [4], signify a departure from the traditional usage of terms such as ‘housing’. This shift raises new critical questions about precisely what constitutes a home. Government policy has historically been built upon the assumptions of policymakers rather than consulting with people who are homeless [22]. This omission created policies shaped by limited or stigmatised understandings of homelessness, leading to supports that were ineffectual, and failing to address the systemic issues that perpetuated homelessness [20, 22]. In more recent times, some reforms have begun to adopt the perspectives of people with lived experience of homelessness, leading to initiatives that prioritise collaboration, a person‐centred approach, holistic support, and tailored solutions to meet the diverse needs of people experiencing homelessness [23, 24].

Home is a physical space that plays a role in a person's psychological and social experience [25]. This physical space is a multidimensional, dynamic and subjective experience, a combination of place and space that results from an accumulation of emotions and experiences that enable ongoing meaning‐making and which shapes identity [26]. Meaningful engagement in personally significant activities that provide purpose and the formation of meaningful daily routines [27] are crucial to a house becoming a home [16, 28]. There is a need to expand the normative notion of home to enhance policymakers’ ability to tailor practices that meet the specific needs of the homeless population and enable more effective transitions out of homelessness. Sustainably accomplishing this ambition requires hearing the voices and experiences of people with lived experience regarding their conceptualisation of home [9, 22, 29].

Some qualitative research has explored the concept of home from the perspective of people who are homeless [25, 26, 28, 30, 31, 32, 33, 34] but these findings remain fragmented across disciplines in small‐scale studies. This study aimed to synthesise existing qualitative research into a collective summary that will strengthen the evidence base and knowledge about the concept of home from the perspectives of people who are homeless. A decision was made to target perspectives of adults aged between 16 and 65 because the structural drivers of homelessness and available health and social care systems differ for people under 16 and people over 65. The study sought to answer the question: *How do people who have experienced homelessness between the ages of 16‐65 describe the concept of home?*

## 2 Materials and Methods

### 2.1 Study Design

This review utilised a meta‐ethnographic approach. Meta‐ethnographies combine findings from multiple qualitative studies, leading to new interpretations and the generation of new theories [35, 36]. This process results in rich data that allows us to go beyond separate accounts of a phenomenon with low sample sizes, to a robust dataset that provides a thick description [35].

As the aim was to gain an understanding of what “home” means for this population, home has not been defined. However, our conceptualisation of home includes but is not limited to any space associated with meaningful experiences, where a person has strong emotional affinity through habituated relationships, routines and occupations that offer people a sense of belonging and connection. This broad conceptualisation has been adopted to include diverse descriptions from participants and ways of inhabiting time and space that have not been sufficiently explored historically. This conceptualisation was used as a guide during screening.

This review was registered with the International Prospective Register of Systematic Reviews (PROSPERO CRD42023493077) before commencing the search. The protocol followed the eMERGe reporting guidelines customised for meta‐ethnographies [37] and followed the PRISMA guidelines for systematic reviews and meta‐analyses, ensuring a rigorous and transparent approach to the research process [38].

### 2.2 Search Strategy

A database search strategy was developed using the SPIDER framework in collaboration with an academic librarian. The sample (S) consisted of people with lived experience of homelessness (either at the time of the study or in their past), the phenomenon of interest (PI) and evaluation (E) was that the research described views, beliefs and experiences about the concept of home, any qualitative study designs (D, R) were included, including mixed methods where the qualitative data could be extracted. The strategy was developed and finalised in Medline before being adapted for other databases, including PsycInfo, EMCARE, CINAHL, ProQuest (encompassing Social Science Database, Sociological Abstracts, and Sociology Database), Scopus, and Web of Science. Search parameters were set to include only English‐language publications, with no limits on the year of publication. The search strategy used for Medline is provided in Supporting file 1. Grey literature was searched through Google, Australian Housing and Urban Research Institute, Analysis & Policy Observatory, Trove, and various homelessness advocacy organisations. The search was conducted on 30th March 2025.

### 2.3 Study Selection

Results were imported to Endnote [39] where automatic and manual duplicate detection was completed. Results were then exported to Covidence [40] where the remaining duplicates were removed. All records were then screened by two researchers at title and abstract and then at full text with reasons for exclusion at full text recorded. LT screened all papers with GRH, BS and CMM sharing the dual screening. Conflicts during title and abstract and full text were resolved through team meetings where a final decision was made.

### 2.4 Inclusion and Exclusion Criteria

Studies were screened against predefined inclusion and exclusion criteria as outlined in Table 1. Studies were included where most participants met the age criteria. For studies that did not report the age range, efforts were made to contact the authors to obtain this information. If authors did not respond or if age data remained unavailable, studies were included in the review as participants were likely to fall within the specified age range and the researchers preferred not to exclude valuable data. Instances where this occurred were noted in the description of the participants. Studies that explored the experiences of internationally and internally displaced populations, such as migrants and refugees, were excluded. While these populations may experience decreased feelings of home, their nuanced experiences were beyond the focus of this review.

**Table 1:** Inclusion and exclusion criteria when screening.

Inclusion	Exclusion	Justification
No limit on publication date	Not applicable	Being as inclusive as possible
Qualitative and mixed methods with any design (e.g., ethnography, surveys with open questions, descriptive qualitative, photovoice)	Quantitative research; systematic reviews; discussion papers or opinion pieces; conference abstracts	Meta‐ethnography only includes qualitative research
Participants who have experienced any form of homelessness between the ages of 16 and 65	Participants outside of this range and studies with wide age ranges (e.g., 30‐60) where separate data is not able to be obtained	Narrowing the age range offers more focused findings to inform policies – people under 16 and over 65 have different structural drivers and health and social care systems
Studies published in English	Studies in languages other than English	The review team did not have access to translators and had no means of checking accuracy of online translation
All countries, cultures, genders	Not applicable	To generate a comprehensive description of home
Studies that feature the concept of home as described by participants in the research and the authors	Studies that did not focus on the meaning of home or did not describe home	Seeking broad and diverse descriptions of home whilst staying within review scope
	Internally and internationally displaced people (i.e., refugees) and cultural homelessness	Beyond the scope of this review

### 2.5 Critical Appraisal of Selected Papers

Given the nature of the research topic, studies being screened originated from diverse health and social researchers meaning there was variability in the methodological detail provided. For this reason, it was decided that screening was needed to check reporting adequacy prior to critical appraisal. This screening was completed using the Reporting Assessment Checklist by Carroll, Booth and Lloyd‐Jones [41] which uses four criteria to assess the transparency with which methods are reported. In keeping with the checklist, it was decided that studies which clearly met two or more of the checklist criteria were adequately reported for inclusion. At first, the decisions about which studies met the checklist criteria were completed by the whole research team. Once a clear interpretation of the criteria was established, the primary researcher (LT) took the lead and independently excluded inadequately reported articles. Included studies then had their procedural rigour appraised in duplicate using the Critical Appraisal Skills Programme (CASP) [42]. Team meetings were held to resolve discrepancies. The CASP was not used to exclude articles based on quality, but to identify and summarise the quality of the papers.

### 2.6 Data Extraction

Characteristics of all studies were extracted independently by two reviewers into a Microsoft Word document [37, 43] including the year of publication, authors, country of data collection, study aim, study design, data collection method, analysis method, sample size, age and gender of the participants [37, 43]. Initial extraction of study findings was done in duplicate for five studies. Meetings were held to compare the extracted data, review consistency and accuracy, and agree on the principles and processes to follow. This established a consistent procedure for extracting data from all subsequent articles, which was carried out by LT. Extracted data included participant direct quotes. Where possible, only quotes from people in the eligible age range were extracted; however, when ages were not provided, then all quotes were extracted. Relevant author interpretation of data in the findings and discussion [35, 43] were also extracted. Where studies that had photos in addition to interview data, only the interview data were extracted. During extraction, close critical reading of studies was performed to ensure consistent extraction and interpretation of data [35, 37, 43].

### 2.7 Data Synthesis

Following extraction, first‐order analysis included coding segments of data in Microsoft Word and then manually sorting these segments into groupings based on common concepts across the studies [43]. For example, coding segments from studies that referred to safety and security were grouped together. The coding was completed in duplicate for five studies, and the manual sorting was completed by all members of the research team during a meeting. Second‐order analysis involved creating a written summary of the initial groupings of data, to support familiarity in data and advance the synthesis. Continuing with the safety and security example, this written description summarised and captured all the different examples, expectations and descriptors used by participants about safety and security across the studies. Both relational and refutational data were included within the groupings (e.g., data describing surveillance both positively and negatively was described in the same summary) [35, 43]. Once groupings were summarised, they were compared and discussed during team meetings to find conceptual translations across the groupings to allow new third (or higher) order themes to form [43]. The third‐order themes provided a line of argument that described the concept of home from the perspectives of people experiencing homelessness [35, 43]. Feedback was provided on these findings by an external consumer representative who was a researcher and expert in homelessness programmes and systems with experience working in a homeless support service. Her professional and research expertise allowed practice‐informed insights that strengthened the way the findings were reported.

### 2.8 Rigour

The eMERGe reporting guidelines for meta‐ethnography were used throughout the review process. The primary researcher maintained a reflective journal to manage any biases or assumptions. Reflexivity was maintained through the lead researcher (LT) keeping a reflective journal and regular team discussions enabled questioning and exploration of how data were being interpreted. As each member of the team had differing views and perspectives about homelessness, this process was applied at each order of analysis to ensure interpretation remained grounded in the data. Additionally, the use of the PRISMA checklist enhanced transparency in the study selection process.

## 3 Findings

### 3.1 Search Outcomes

**Figure 1:**
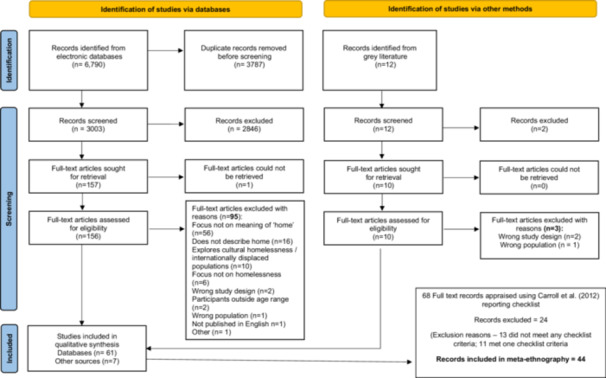
PRISMA flow diagram.

There were 6,790 records identified from data base searching. Following duplicate removal, 3003 records were screened at title and abstract, resulting in 2,846 records being removed. This led to 156 records being screened at full text and inclusion of 61 records. The grey literature search led to inclusion of a further seven sources and 68 overall. Full details are provided in the PRISMA flow diagram (Figure 1).

### 3.2 Study Characteristics

The review included 44 papers, conducted in 10different countries. The countries included Canada (*n* = 14), the United States of America (*n* = 12), the United Kingdom (*n* = 5), Australia (*n* = 5), New Zealand (*n* = 2), Ireland (*n* = 2), and one paper from Denmark, Finland, Italy, and the Philippines. Two articles collected data from participants in two different countries [16, 44]. Collectively there were 1023 participants; although this is higher as one study did not report the number of participants [45]. Of those participants whose gender was specified, there were 464 female, 499 male, either transgender and/or non‐binary (*n* = 18), transgender female (*n* = 2), transgender male (*n* = 5), and one participant declined to report a gender [46] and three papers did not report gender [45, 47, 48]. Across the included studies, there were five that did not report participant age [47, 48, 49, 50, 51], and three reported age ranges that exceeded the maximum age limit and did not differentiate data to individuals [52, 53, 54]. As the participants over 65 are likely to be minimal, these studies were interpreted using the same methods as the other 36 studies.

Nine studies were phenomenological [50, 54, 55, 56, 57, 58, 59, 60, 61], eight were ethnographic (case study, symbolic interactionism) [21, 47, 48, 49, 62, 63, 64, 65], five were grounded theory (constructivist) [16, 52, 66, 67, 68], six were qualitative descriptive [44, 69, 70, 71, 72] four were narrative enquiry [45, 51, 73, 74], four were participatory research [33, 67, 75, 76], three did not report design [4, 77, 78], two were mixed methods [46, 79], and there was one study each of, photovoice [53], appreciative enquiry [80], and interpretivist [81]. Studies mainly collected data through various forms of interviewing (face‐to‐face, ‘go‐along’, in‐depth, narrative, open‐ended, photo‐elicitation, semi‐structured, and unstructured interviews) [4, 16, 21, 33, 44, 45, 46, 47, 48, 49, 50, 51, 54, 55, 56, 57, 58, 59, 60, 61, 63, 64, 67, 71, 72, 73, 74, 75, 76, 77, 78, 80, 81, 82], other methods included photography [33, 48, 51, 53, 56, 57, 58, 62, 65, 76], participant/fieldwork observations [4, 16, 21, 47, 49, 55, 59, 60, 62, 63, 66, 73], workshops [33, 76, 80], surveys (qualitative and quantitative) [44, 46, 79], focus groups [53, 63], creative writing, design charrette, and digital storytelling [33, 76]. There are three instances of both a dissertation and an article from the same research project being included [33, 56, 57, 59, 60, 76]. A summary of the study aims, study designs, data collection and analysis methods and participant details for each study is provided in Table 2.

**Table 2:** Study characteristics.

Author, year country	Study aim	Study design	Data collection method	Data analysis method	Number^A^ and gender	Age^B^
Baumann [55], 1993 USA	The meaning of being homeless for women in homeless families	Phenomenology	SSI, observations, fieldnotes	Inductive thematic Analysis	15 f	20–36 years
Bell [49], 2015 Canada	How social worlds overcome stigma when homeless	Ethnographic (using symbolic interactionism)	Participant observations, in‐depth interviews	Coded and organised thematically	10 m	Not available^C^
Boland [16], 2023 UK & Ireland	The occupational processes to sustain tenancies for homeless people	Constructivist Grounded theory	SSI based on photo‐elicitation. Primary researchers’ notes, memos and journal	Line‐by‐line coding, focused coding, memos, mapping and theoretical sampling	29; 5 f, 24 m	20–65 +^D^ years
Brown [83], 2005 USA	Tenants experiences in supported housing	Grounded theory	In‐depth SSI	Thematic analysis (using open coding)	8; 4 f, 4 m	33–50 years
Brueckner [77], 2011 Australia	Transition into permanent, independent accommodation	Not Reported	In‐depth face‐to‐face interviews	Interpretive phenomenology (combined with discourse analysis)	19; 15 f, 4 m	16–22 years
Burns [66], 2016 Canada	The meaning of place for first time homeless	Constructivist Grounded theory	SSI, field notes/observations	Constant Comparison (with theoretical sampling)	15; 7 f, 8 m	50–80^D^ years
Burns [52], 2020 Canada	Experiences of home in permanent supported housing	Case Study & Constructivist Grounded Theory	In‐depth SSI	Constructivist Grounded theory	10 m	55–77 years
Burns [67], 2022 Canada	Sense of place for homeless people once they are housed	Participatory Research (part of a larger study)	“Go‐along” SSI	Thematic analysis	7; 4 f, 3 m	55–67^D^ years
Canham [75], 2023 Canada	“Ageing in the right place” after being homeless	Community‐based participatory research (part of a larger study)	Photovoice and in‐depth SSI	Inductive Thematic analysis	11; 6 f, 5 m	58–75^D^ years(Mean age 65)
Chan [69], 2020 USA	Creation of home and community for former homeless	Qualitative	SSI based on drawing activity	Thematic analysis (using open coding)	37; 20 f, 17 m	28–65 years
Coward [81], 2018 England	Experiences and perceptions of home for former homeless	Interpretivist	SSI	Inductive thematic analysis	17; 5 f, 12 m	18–49 years
Farrugia [78], 2011 Australia	Contribute to literature on youth homelessness	Not Reported	Interviews	Open and theoretical coding	20; 9 f, 11 m	16–24 years
Gaboardi [53], 2018 Italy	Descriptions of home from perspective of homeless	Photovoice	Photovoice, photo‐elicitation group discussions	Constant comparison (from grounded theory)	5 m	47–67 years
Groot [62], 2012 New Zealand	How events and relationships influence life for homeless peoples	Ethnographic case study	Fieldwork observations, interviews and photo‐voice	Not Reported	1 m	48 years
Henze‐ Pederson [4], 2024Denmark	Explore the meaning of home for women and children escaping violence	Not reported – combined data from 2 studies	fieldwork, interviews, observation	Merged data and then categorised before doing thematic analysis	First study ‐ 26 fSecond study ‐ 14 f	24–49 years
Hoolachan [63], 2022 Scotland	Engaging in place‐making practices within supported hostels	Ethnography	Participant observations, SSI, focus group, fieldnotes	Sequential approach	22; 6 f, 16 m	16–21 years
Iaquinta [54], 2017 USA	The meaning behind creating home for former homeless	Hermeneutic phenomenology	SSI	Van Manen's four stages (Phenomenology)	14; 5 f, 9 m	23–69years
Isenhower [64], 2003 USA	The experiences of homeless mothers living in a shelter	Ethnography	SSI	guided by feminist and development psychology	15 f	20–42 years
Julien‐Chinn [45], 2022 USA	Resilience and protective factors among former houseless families	Qualitative Narrative	Narrative interviews	Deductive Thematic Analysis	Number of participants and gender not reported^E^	All over 18^E^ years
Kidd [44], 2011 Canada/USA	The meanings attributed to the concept of home and impact on resilience	Qualitative	SSI, Survey	Content analysis (open coding and organised thematically)	208; 84 f, 122 m	14–24 years (Mean age 20)
Kirkpatrick [73], 2009 Canada	Experiences of exiting homelessness and into stable housing with support	Narrative Enquiry	ObservationOpen‐ended, in‐depth Interviews	Reading, Analysing and re‐telling stores (Three‐dimensional enquiry space)	12; 6 f, 6 m	19–52 years
Langegger [47], 2016 USA	Relationships between structural conditions and behaviour of homeless people	Ethnography	Interviews, field notes, observations	Coding of all interviews and field notes	40^E^; Gender not available^E^	Not available E
Mace [80], 2023 New Zealand	Identify effective transition strategies to sustaining a permanent home	Appreciative Enquiry	In depth interviews using AI's 4‐D's, 2 workshops	Reflective thematic	20; 5 f, 6 m	< 10–59 years
Manson [21], 2024 Canada	The contexts that shape movements in and out of homelessness	Ethnography	Semi‐structured interviews, follow‐up interview, fieldwork	Coded using ethnographic and participatory action research framework	54^G^ cis 46, 6 t, 2 nb, 19 f, 33 m	19–29 years
McCarthy^F^, 2015 [56] & 2020 [57] England	How women negotiate their identities following homelessness	Phenomenological visual methodologies	In‐depth interviews, participant‐led photography, photo‐elicitation	A priori and inductive thematic analysis informed by grounded theory	12 f	18–49 years
McDonell [50], 2014 USA	The experiences of the houseless population in Hawaii	Phenomenology	In‐depth SSI	Thematic analysis using line‐by‐line coding and categories	16; 10 f, 6 m	Not available^C^
Merza [58], 2023 Philippines	The interpretation of home by seven gay homeless men	Interpretive phenomenological	Photovoice, SSI	Framework analysis	7 m	28–31 years
Milligan [46], 2024 USA	How adults living in permanent supportive housing describe their concept of ‘home’	Longitudinal mixed methods	Mixed methods survey, interviews data collected at 12 months [T2] and 18 months [T3]	T2 and T3 combined in thematic analysis; ontological security as analytical framework	28; 10 f, 12 m, 5nb/t, 1 declined to respond	19–40 years (Mean age23.2)
Nousiainen [51], 2021 Finland	Experiences of home and housing for formerly homeless	Narrative approach	Narrative “Go‐along” interviews and photovoice	Content Analysis	11; 6 f, 5 m	Not available^C^
Osuji^F^, 2010^59^ & 2015 [60] Canada	Exiting homelessness for women without children	Hermeneutic phenomenology	interviews, participant observations	Thematic analysis	12 f	34–65 years
Padgett [70], 2007 USA	Describing ‘home’ and ontological security for those in permanent housing	Qualitative Descriptive	Two life history interviews with each participant	Constant Comparative Analysis from Grounded Theory	39; 13 f, 26 m	Mean age 48 years
Parsell [79], 2015 Australia	Experiences of community in supportive housing	Mixed Method	Survey (Qualitative and quantitative)	Qualitative‐ iterative analytical process	120; 62 f, 58 m	20–70^D^ years
Parsell [71], 2016 Australia	Maintaining control over lives under continual surveillance	Qualitative	In‐depth interviews	Thematic analysis; inductive process	28; 6 f, 22 m	25–65^E^ years
Phipps [61], 2017 England	Perspectives about psychologically informed environments	Phenomenological epistemological approach	SSI	Thematic analysis	9; 1 f, 8 m	46–55 years
Plage [48], 2025Australia	The perspective of the former homeless	Oikography/Ethnography	Photographs, interview, digital storytelling	Narrative dialogic/performative analysis	14; gender not stated	Not stated
Quilty [74], 2022 Ireland	Links between sexual and gender identities and meanings of housing and home	Narrative	In‐depth Interviews	Pathways to Home Framework	22; 10 cis f, 1 cis m, 1 nb/q, 1 t/hum, 3 nb, 2 nb/t, 2 t, 1 t/m, 1 t/f	18–30 years
Robertson [65], 2007 Canada	Homeless women with HIV negotiating identity and home in stigmatised space	Ethnography	Open‐ended interviews and ‘walk‐about’ taking photos	Not Reported	14 f	32–49 years
Tsai [68], 2020 USA	How homeless veterans define and conceptualise home and homelessness	Grounded theory	SSI	Inductive thematic analysis	19; 3 f, 16 m	49–67 years (Mean age 57.83)
Walsh^F^, 2009 [33] & 2010^76^ Canada	The characteristics of home for women who are, have been or are at risk of being homeless	Participatory (Action) Research	Digital storytelling, interviews, photovoice –creative writing & design workshops	Conventional content analysis with grounded theory approaches	20 f	22–64 years
Wood [82], 2024Canada	How homeless describe their futures and expectations	Qualitative descriptive approach	Semi‐structured interview	Thematic analysis	38; 15 cis f, 17 cis m, 4 t/m, 1 t/f, 1 nb	Mean age 19.84 years
Woodhall‐Melnik [72], 2017 Canada	Housing first programmes and the concept of home and housing stability	Qualitative	SSI	Coding used to establish final themes	15 m	39–63 years (Mean age 51)

Abbreviations: AI, appreciative enquiry; cis, cisgender; f, female; hum, human; m, male; nb, non‐binary; q, queer; SSI, semi‐structured interviews; t, transgender; UK, United Kingdom; USA, United States of America.

^A^ Number of homeless participants only.

^B^ Age at time of study.

^C^ Could not contact author.

^D^ Individual ages reported in‐text, only quotes from individuals within age range extracted.

^E^ Information provided by author.

^F^ Article and dissertation based on the same study.

^G^ Participants’ gender identified in more than one way.

### 3.3 Critical Appraisal Findings

Some studies inadequately reported study methods/characteristics, which hindered the contextualisation of the findings [35] required for synthesising. As a result, 24 records were excluded in the first appraisal round, leaving the final 44 (see Figure 1 for exclusion reasons). The remaining studies were appraised using the CASP with the majority meeting the criteria for stated aim and qualitative purpose. Across the studies, there was often insufficient reporting of participant recruitment and a lack of reflection on the researcher‐participant relationship. There were some instances of papers recording a ‘no’ or ‘can't tell’ for ethical approval which appeared to be due to study designs being ethnographic/field immersion and/or inadequate reporting in the research. These papers were still included as the research is publicly available and published. An overview of the critical appraisal findings can be found in Table 3.

**Table 3:** Qualitative critical appraisal findings with CASP.

Author and year	Aim	Purpose is qualitative	Research design	Recruitment	Data collection	Researcher relationship	Ethics	Data analysis	Findings
Baumann, 1993 [55]	Y	Y	Y	CT	CT	CT	N	CT	Y
Bell & Walsh, 2015 [49]	Y	Y	Y	Y	Y	CT	Y	CT	Y
Boland, 2023 [16]	Y	Y	Y	Y	Y	Y	Y	Y	Y
Brown, 2005 [83]	Y	Y	Y	Y	Y	CT	CT	Y	Y
Brueckner, 2011 [77]	Y	Y	CT	CT	Y	CT	Y	CT	Y
Burns, 2016 [66]	Y	Y	Y	CT	Y	CT	CT	Y	Y
Burns, 2022 [67]	Y	Y	Y	CT	Y	N	Y	Y	Y
Burns, 2020 [52]	Y	Y	Y	Y	Y	CT	Y	Y	Y
Canham, 2023 [75]	Y	Y	CT	Y	Y	N	Y	Y	Y
Chan, 2020 [69]	Y	Y	CT	CT	Y	CT	Y	Y	Y
Coward, 2018 [81]	Y	Y	Y	Y	CT	CT	CT	N	Y
Farrugia, 2011 [78]	CT	Y	CT	Y	Y	CT	N	CT	CT
Gaboardi, 2018 [53]	Y	Y	Y	CT	Y	Y	CT	Y	Y
Groot, 2012 [62]	CT	Y	Y	CT	CT	CT	N	N	Y
Henze‐Pederson, 2024 [4]	Y	Y	CT	Y	CT	N	Y	N	Y
Hoolachan, 2022 [63]	CT	Y	Y	CT	Y	CT	Y	Y	Y
Iaquinta, 2017 [54]	Y	Y	Y	Y	Y	CT	Y	Y	Y
Isenhower, 2003 [64]	Y	Y	Y	Y	Y	Y	Y	Y	Y
Julien‐Chinn, 2022 [45]	Y	Y	Y	CT	CT	Y	Y	Y	Y
Kidd, 2011 [44]	Y	Y	CT	Y	Y	CT	Y	Y	Y
Kirkpatrick, 2009 [73]	Y	Y	Y	Y	Y	Y	Y	Y	Y
Langegger, 2016 [47]	CT	Y	Y	CT	CT	N	N	CT	Y
Mace, 2023 [80]	Y	Y	Y	Y	Y	Y	Y	Y	Y
Manson, 2024 [21]	Y	Y	Y	Y	Y	N	Y	Y	Y
McCarthy, 2020 [57]	Y	Y	Y	CT	Y	Y	CT	N	Y
McCarthy, 2015 [56]	Y	Y	Y	CT	Y	Y	Y	N	Y
McDonell, 2014 [50]	Y	Y	Y	CT	CT	CT	Y	Y	Y
Merza, 2023 [58]	Y	Y	Y	Y	Y	N	Y	Y	Y
Milligan, 2024 [46]	Y	Y	Y	Y	Y	Y	Y	Y	Y
Nousiainen, 2021 [51]	Y	Y	Y	Y	Y	N	Y	Y	Y
Osuji, 2010 [59]	Y	Y	Y	CT	Y	Y	Y	Y	Y
Osuji, 2015 [60]	CT	Y	CT	CT	CT	CT	Y	N	Y
Padgett, 2007 [70]	Y	Y	Y	Y	Y	N	Y	Y	Y
Parsell, 2015 [79]	Y	Y	CT	Y	Y	N	N	CT	Y
Parsell, 2016 [71]	Y	Y	CT	CT	Y	N	Y	CT	Y
Phipps, 2017 [61]	Y	Y	CT	Y	CT	N	Y	Y	Y
Plage, 2025 [48]	Y	Y	Y	N	Y	N	Y	CT	Y
Quilty, 2022 [74]	Y	Y	N	Y	CT	Y	Y	N	Y
Robertson, 2007 [65]	CT	Y	CT	CT	CT	CT	CT	N	Y
Tsai, 2020 [68]	Y	Y	CT	CT	Y	N	Y	Y	Y
Walsh, 2009 [33]	Y	Y	Y	CT	Y	Y	Y	CT	Y
Walsh, 2010 [76]	Y	Y	Y	CT	Y	N	Y	Y	Y
Wood, 2024 [82]	Y	Y	Y	Y	Y	N	Y	Y	Y
Woodhall‐Melnik, 2017 [72]	Y	Y	N	CT	CT	N	CT	CT	Y

Abbreviations: CT, cannot tell; N, no; Y, yes.

## 4 Meta‐Synthesis Findings

Following second order analysis, there were six overarching themes with 70 subthemes. Further synthesis of the data constructed three final themes, each with several subthemes. These themes were: ‘home is stability and return to self’, ‘home is a protective and controllable threshold,’ and ‘home is having connection’. Table 4 highlights which papers contributed to each subtheme. Quotes presented in Table 5 illustrate each theme and subtheme.

**Table 4:** Papers contributing to each theme and subtheme.

Themes and subthemes		No.	References
Home is stability and return to self	Having a consistent foundation	23	[4, 21, 33, 46, 47, 48, 53, 54, 55, 56, 57, 60, 64, 67, 69, 72, 75, 77, 78, 81, 82, 83]
	Personalising the environment	31	[4, 16, 21, 44, 46, 47, 48, 51, 52, 54, 55, 56, 57, 58, 62, 64, 65, 69, 71, 72, 73, 76, 78, 79, 80, 81]
	Finding yourself	18	[4, 16, 44, 46, 53, 54, 55, 56, 58, 59, 60, 62, 65, 69, 70, 72, 78, 81]
	Dreaming of the future	17	[21, 46, 48, 51, 53, 54, 56, 58, 59, 62, 63, 64, 70, 73, 80, 81, 82]
Home is a protective and controllable threshold	Exerting emotional and physical boundaries	11	[4, 16, 21, 46, 48, 54, 65, 76, 78, 80, 83]
	Having a personal retreat	36	[4, 33, 44, 46, 48, 49, 50, 51, 53, 54, 55, 56, 58, 61, 62, 63, 65, 66, 67, 68, 69, 70, 71, 72, 73, 74, 76, 77, 78, 80, 81, 83]
	Establishing ownership	13	[46, 47, 62, 63, 68, 69, 72, 73, 80, 81, 82, 83]
	Enacting self‐determination	30	[4, 16, 21, 33, 44, 46, 50, 51, 52, 53, 55, 56, 57, 59, 62, 63, 65, 66, 67, 68, 69, 70, 71, 72, 73, 75, 77, 80, 81, 83]
Home is having connection	Connecting to something more	26	[16, 33, 44, 45, 46, 50, 51, 52, 54, 55, 56, 57, 58, 59, 60, 62, 63, 64, 66, 68, 69, 72, 75, 80, 81, 82]
	Finding your people	29	[4, 16, 21, 44, 46, 49, 50, 52, 53, 54, 55, 56, 58, 59, 60, 62, 63, 64, 65, 66, 67, 68, 69, 71, 73, 74, 77, 80, 81]
	Feeling loved	24	[4, 21, 33, 44, 49, 50, 51, 52, 53, 55, 56, 58, 59, 60, 61, 62, 66, 67, 68, 71, 73, 80, 81]
	Experiencing community connection	21	[16, 21, 44, 47, 49, 52, 53, 54, 55, 56, 62, 63, 66, 67, 68, 69, 70, 71, 74, 77, 80]
	Engaging in routine and occupations	25	[4, 16, 21, 33, 44, 47, 48, 51, 52, 54, 56, 59, 63, 66, 67, 70, 71, 73, 77, 80, 81, 82, 83]

**Table 5:** Illustrative quotes for themes.

Theme	
Home is stability and return to self	Quote
**Having a consistent foundation**	*“I am just starting to feel like I have a home. Home is like a base…you get secure enough in your base or your foundation…the things that can happen are limitless”* [76] ^*p. 201*^ *“stability and knowing where you are staying every night is calling somewhere home”* [81] ^*p. 131*^ *“The house is essential … Home is were[sic] we stabilise”* [53] ^*p. 56*^ *“when I first moved into my place I was living out of a cardboard box. I refused to unpack… And then I finally got to a place where I was like, okay, I'm going to be here for a while. I can unpack. And I, like, set my stuff up. And I was like home”* [21] ^*p. 892*^
**Personalising the environment**	“My home is somewhere where I've got my stuff in, it's… decorated as how I want it” [57] ^p. 1322^“Just having all my things in the apartment, even though it is a bit small makes it home” [79] ^p. 1198^“A home should be an indication of who you are. It should represent you and yourself. When you don't have a home, you really don't have an identity, because you don't have anything that represents yourself” [44] ^p. 761^“I've started getting plants, which I haven't had for years because plants need a place to sit and grow. They really do like to stay in the same place for a year or two before they start really branching out… life is like that, once you take root and you start sprouting off in all different directions … and you smell nice” [48] ^p. 5^
**Finding yourself**	“So, what I've really liked [in the new place] is that it's been me all the way… I decided where things should be and how the pictures should hang and things like that. It's just me”. [4] ^**p. 541**^“And you need a spot where you can lock the door. So that you can start piecing this back together…Like all of it back together, your ID” [72] ^p. 370^“Because I've not got anywhere to live, I don't feel a complete person” [56] ^p. 185^
**Dreaming of the future**	“I am part way out of the pit of homelessness…What this place is to me – it's a place where you can dare to dream again” [54] ^p. 87^“Honestly, like my number one goal is stability. Like a career, home, regular routine. Like that's my like, dream” [82] ^p. 63^
**Home is a protective and controllable threshold**	
**Exerting emotional and physical boundaries**	“Nobody's getting into the room, only the people I know…As for street people, no, nobody's going to know where I live” [65] ^p. 544^“I would desperately like my own home, somewhere where I can close the doors” [62] ^p. 262^ [58] ^(p.262)^“It feels like home because not many people know where I live” [78] ^p. 369^
**Having a personal retreat**	“Somewhere safe and secure where I could go and I had control over who was going to walk to my door and who wasn't” [71] ^p. 3198^“The impact of not having your own space, like a space that you can exist alone and not have to deal with anyone or be expected to perform in any way…The effect that has on you…is really exhausting” [74] ^p. 13^“This is my home…No one can come here if I don't let them. I command this threshold” [51] ^p. 72^“Home for me is a safe place” [80] ^p. 133^“My home is just me and my family and my kids, and there's that line between us and the rest of the world, you know?” [33] ^p. 309^
**Establishing ownership**	“It feels good to be able to go home and put that key I'll never forget that feeling of having my own set of keys” [68] ^p. 111^“It's mine. It's actually my home now, I can make it mine. I've made it mine… It's just the fact that it's mine that makes it feel like home” [81] ^p. 125^
**Enacting self‐determination**	“I feel better, I eat what I want. I eat when I want. If I get up at night and want to eat ham, I'll eat it” [52] ^p. 298^“Ultimately it's…where I can do what I want, cook, relax, and just live like a normal person again” [75] ^p. 7^“Without a home, you can't do anything about your life. You don't have any control…its like they are trying to take your determination away” [55] ^p. 66^“It [having a home] lets me do what I want to do when I want to do it. You know I don't have to answer to anyone…having your own place is like having a clubhouse or something. I am king of the hill” [83] ^p. 54^“What I call “home,” home. Well I'm the leader, or how you say it, “I'm the captain of my ship” [69] ^p. 109^
**Home is having connection**	
**Connecting to something more**	“I think houseless is a better term. Especially for the population we're talking about, Native Hawaiians, Micronesian, because the land, this is our home” [45] ^p. 471^“No this is my home, I'm just houseless. That's what I mean, we don't have a roof over our head, we don't have a shelter but this is home. Hawaii is home” [50] ^p. 78^“To me, a home is where you lay your head…That's what you call home. And for my people, my Native people, home used to be the forest. That's what they called home” [44] ^p. 765^
**Finding your people**	“The human being is social from birth, the friends are home” [53] ^p. 57^“The shelter is a necessity. But you know, our family's, we've always been a home as long as we've got each other” [64] ^p. 52^“because I have a family now and a son and a fiance. I've gotten close with some of the residents here, so yeah it feels like home” [46] ^p. 1092^
**Feeling loved**	“I got a family here…I said to one of the service providers, you're like a sister to me” [66] ^p. 16^“It's nice to go home at the end of the day to people who love and care about you…that you matter and give a sh*t that you come home at night” [49] ^p. 1986^“Home is a place where you are loved, where you are cared for, where there is laughter. … To be home is being happy, being loved, being content, being cared for by your parents, or by whoever you live with” [59] ^p. 93^
**Experiencing community connection**	“It feels like a home to you and you know the staff, you know everyone…moving out from there…[it's] quite difficult” [61] ^p. 32^“I have to either get myself a job, a volunteer position, or something… I have to be doing something constructive…the goal is to reintegrate you back into society” [70] ^p. 1932^
**Engaging in routine and occupations**	“Home feels like – I can walk into the sitting room now, turn on the telly and lie there, then look at my phone…that's the real me like I remember” [16] ^p. 1248^“At night we'd always have a campfire. We'd work all day, …we'd get our money together and go get…food…We'd sit around the fire telling stories, laughing, just hanging out. We'd have music…It was a ritual” [47] ^p. 651^“Oh yes, I feel at home like I said I have my own room, my own TV, my own computer, got my books, my little routine” [67] ^p. 6^

### 4.1 Home Is Stability and Return to Self

This theme includes four subthemes that describe how a consistent, stable place to keep and enjoy possessions rebuilt identity [72] and self‐worth. It was a starting point for dreaming [53] and hoping [53, 54, 80] for better days [80] and “learning how to live.” [70] ^(p.1932)^ A stable shelter enabled participants to feel whole [59] and to develop foundations [69, 83].

#### 4.1.1 Having a Consistent Foundation

People living in temporary [46, 48, 54, 57, 59, 64, 69, 75, 81] unstable [64, 72, 81] dwellings often exhibited emotional detachment [48, 69, 81] to the place. It was just somewhere to live, sleep [81] and return to after work [67, 81], a stepping stone [46, 72, 78, 81] towards something better [54, 81]. Some had become accustomed to instability [77, 81] and no longer sought a permanent home. The constant moving and starting over was exhausting [4, 55, 56, 69] often resulting in the loss of possessions [4, 21, 56]. Instability and precariousness of housing sometimes led to differences in the data between private, public, shared housing and home ownership [48]. Home [46, 72, 81] was established through having predictable [21], reliable [33, 46], secure [46, 59, 76, 81], stable [72, 77, 81, 82], consistent [46, 81], permanent [77, 81] shelter, which served as a base for life [33, 53, 69, 76, 81, 83]. Some felt they needed to resolve personal issues to feel stable at home [72]. Even in permanent housing, some still feel unstable, as if they are living close to homelessness [69], or experience complicated feelings about the markers of a consistent home [46].

#### 4.1.2 Personalising the Environment

Personalisation [16, 47, 48, 57, 62, 68, 81, 83] of environments through possessions transformed spaces into familiar [16, 63], comfortable [69], secure [48], homelike [16, 44, 46, 54, 55, 56, 57, 61, 68, 69, 71, 72, 76, 78, 81, 83], places. Whether items were past [4, 56, 57, 62, 63, 68, 72, 81] sentimental possessions or newly acquired essentials [4, 47, 52, 54, 55, 62, 64, 69, 73, 76, 78, 80, 81], they elicited positive emotions [16, 83] such as pride [52] and hope [54, 57]. Through personalising [81] their environment people expressed themselves [21, 44, 54, 56, 58, 68] and their identity [16, 44, 54, 56, 57, 58, 61, 68, 72, 81], providing a tangible link to their past [51, 57, 62]. This connection to personal history [51, 57, 62] strengthened emotional ties to materialised space [16, 21, 48], helping people stabilise and rebuild their identity and belonging [56, 57, 62, 63, 68, 72]. Possessions synonymous with family helped preserve memories [56, 57, 63] maintained past connections [56, 57, 63, 68] and provided emotional support when feeling low [56, 57, 81]. Sentimental possessions included such things as family photographs [56, 57, 63], heirlooms [68, 81], ornaments [56, 57, 62, 63], and posters [56, 57]. It was challenging to accumulate possessions when living transient lives [16, 71] due to barriers such as living out of a single‐bag [81], carrying all their items [55, 69, 71, 81], theft [16, 52, 55, 57, 62, 81], lack of storage [54, 69], limited finances [46], abandoning possessions [65], and loss of items [16, 55].

#### 4.1.3 Finding Yourself

Homelessness could lead to a loss of selfhood [44, 56, 59, 60, 62, 65, 69, 78], and self‐respect [55, 59, 62], causing some to feel alienated [4] or incomplete [46, 56]. In contrast, those with street identities [16, 57, 65] struggled to let go of them [16, 57, 65]. Finding home equated to finding identity [44, 46, 56, 59, 72] through the stability [70], freedom [4, 56] and space it provided for reflection [70] and for engaging in regular activities [69]. Reflection and engagement in activities aided the repair [56, 59, 69, 70] of fragmented past identities and the development of new ones [44, 69, 78]. Home was described as a therapeutic space [72], that fostered dignity [4, 53, 55, 59], confidence [59, 69], self‐esteem [44, 59], self‐respect [44], pride [44], positive selfhood [81], and personal fulfilment [54, 58].

#### 4.1.4 Dreaming of the Future

Typically once people had a home [53, 54, 62, 80, 81], or a dwelling [70, 81] which provided a secure [46, 70, 80] stable [80] foundation [53, 70, 81], they began to look toward the future [46, 53]. This suggests that stable housing allows people to think and plan for the future, and is a ‘big step’ toward achieving dreams [82]. Access to resources and supports [81] provided people with the freedom [59, 70] and hope [54, 59, 62] to dream [54], re‐evaluate [70], set goals [54, 80, 81], and find purpose [70] with a new outlook and meaning for the future [46, 48, 53, 54, 81]. Desire to reconnect with family was common [64], but they often did not know how to do this [46] or was clouded by past negative experiences [51, 56, 73, 81]. Future aspirations are outlined in table 6.

**Table 6:** Future aspirations.

Personal growth	Finding employment [54, 80, 81], pursuing education [21, 54], pursuing hobbies [56, 80]
Health and wellbeing	Recovering from substance use [54], growing old [54], maintaining and staying in their current home [54], saving money for a safe place [46]
Relationships	Marriage [54], reconnect with family/children [54, 56, 64], having a baby [46], adopting a child [58]
Lifestyle	Acquire possessions [48, 54, 63], relocate [54], travel [80], and purchase a home [54, 56, 80] with desired features [56, 58, 80]

### 4.2 Home Is a Protective and Controllable Threshold

This theme describes how home served as a protective barrier [51, 73, 83] from the outside world [33, 44, 52, 55, 63, 69, 71, 72, 74] which was cherished [54, 62, 68]. This barrier was usually a door [21, 33, 48, 51, 62, 72, 73, 81], allowing people to choose who enters their space [33, 51, 55, 63, 65, 69, 71, 73] and what happens within it [44, 46, 63]. Those residing in crisis housing often had limited choice over who could enter their space due to restrictions on visitation which created non home like spaces [46, 55, 63, 69, 71, 73]. Private spaces led to ownership, and autonomy, as well as providing emotional and physical safety which is explained using four subthemes.

#### 4.2.1 Exerting Emotional and Physical Boundaries

When exiting homelessness [54, 78, 83] having a space and physical boundary provided control over reconnecting [54], distancing [46, 48, 54], and disconnecting [54, 78, 80] from relationships [4, 16, 54, 78] and places [76]. Home delineates a boundary around oneself, that if sustained, afforded, safety [46, 48], control [46, 48], and agency [46] over the ‘inside’ and ‘outside’ environment [4, 48]. Having “*a place to go*” [83]^(p.58)^ helped to escape old habits and lifestyles [21], and could support recovery from substance use [21, 76, 80, 83]. To ensure a person's wellbeing [54] and secure their home [78], some severed ties with demanding [16, 54] or untrustworthy [78] people. Disconnecting from ‘bad people’ who attract trouble [78] or a relapse [54, 78] supported the development of a new non‐homeless identity [65, 78], and enabled focusing on one's health [65].

#### 4.2.2 Having a Personal Retreat

Home [44, 54, 56, 69, 70, 71, 72, 74, 78, 80, 81, 83] was a personal space [33, 68, 69, 71, 72, 74, 81] of solitude [54, 72], respite [48], a “*little world*,” [79] ^(p.1198)^ sanctuary [44, 54], “*little bubble”* [46] ^*(p. 1093)*^, haven [44, 69, 70, 80, 83], kingdom [78], castle [81], and fortress [83]. Personal spaces provided them with safety [33, 44, 50, 52, 56, 61, 62, 67, 68, 69, 71, 72, 74, 76, 79, 80, 81, 82, 83], privacy [4, 55, 62, 69, 83], comfort [33, 44, 72, 74, 80, 81, 82], and dignity [52, 53]. For some, this personal space was their apartment [69], or room [81]; even their lazy‐boy chair [80]. This refuge [54, 56, 80, 81] served as a space of solace [83], serenity [54, 69], peace [4, 46, 52, 54, 69], quiet [4, 63, 69, 70], free from the constant stress and vigilance of the streets [46, 48, 56, 62, 66, 70, 83]. This retreat provided feelings of ease [33, 46, 69, 80, 81] away from pain [81], and offered people a secluded emotional space [62] where recovery [4, 54, 61, 80, 83] from past trauma [80], substance use [61, 83], and mental health challenges [83] could begin. It enabled people to “*switch off*,“ [62]^(p. 262)^, have *“peace and quiet”* [4] ^p. 540^ gather their thoughts [54] and foster well‐being practices [44, 48, 72, 80] such as meditation [44, 54], reflection [54, 62], and relaxation [44, 56, 62, 69, 70, 78, 81]. For LGBTQIA+ people, this retreat was a refuge from societal prejudice and discrimination [52, 58, 65, 74] where they could openly express their true identity [52, 74], without having to hide or censor themselves [74], free from fear of judgement or hostility [4, 52, 58, 74]. In this space, people could exert a physical and emotional boundary [33, 46, 48, 52, 56, 69, 70, 73, 81, 83] to recuperate [54, 70, 80] from their interactions with the outside world and manage a balance between private solitude and social interaction [4, 48, 55, 63, 65, 80]. They could choose when to be alone [33, 44, 46, 55, 62, 63, 65, 69, 72, 74, 80, 81, 83]. Surveillance either supported this safe haven or prevented feeling at home. The findings that supported and violated safety, positive and negative experiences of surveillance and the forms of surveillance are outlined in Table 7.

**Table 7:** Details of safety and surveillance.

Safety	
**Findings that supported feelings of safety**	**Findings which violated** [53, 55, 63, 66, 69, 73, 74] **safe personal space**
Positive relationships with crisis housing staff [81] and residents [61, 66]	Negative behaviours [52, 54, 71, 76, 77, 79] of staff [61, 77] and residents [4, 52, 69, 74, 77] in crisis housing. Feeling judged by other residents [4].
Stable housing [70, 75]	Unwell or intoxicated resident of crisis housing [50, 54, 71, 79, 83]
Living in a neighbourhood perceived as safe [69, 75]	Violence and abuse related to sexual orientation [52, 74], racism [52], derogatory attitudes [52, 77] of crisis housing tenants
	Overcrowding issues [66] in crisis housing
	Unwanted phone calls, door buzzers, or unwanted visitors [51, 56]

**Surveillance**	
**Positive experiences**	**Negative experiences**
Providing feelings of relief and protection [71, 79]	Undermined privacy [4, 66, 69, 71, 72, 79, 83]
Staff monitoring helped residents screen and control visitors [65, 79]	Resulted in abnormal [52, 77] and demoralising experiences [69, 77]
Regular checks and monitoring supported and reinforced sobriety efforts [52, 61, 66, 79]	Impacted feelings of autonomy [46, 79], freedom [46, 79], and control [46, 79]
	Created a “*jail like atmosphere”* [79] ^(p.1201)^
	Experienced as invasive [64] and intrusive [79]

**Forms of surveillance**	
24‐h CCTV or watched 24/7 [46, 52, 71, 79]	
Regular and random room checks [52, 69]	
Controlled entry [69, 71, 79]	
Restrictions on visitors [46, 52]	
Searches of lockers and belongings [49]	
Sleeping in shared dormitories [66]	

#### 4.2.3 Establishing Ownership

Having their own space provided the ability to claim ownership to spaces that were theirs [46, 47, 68, 81, 82, 83]. Having a set of keys [68, 72, 73, 77, 83] represented freedom [68], pride [68], joy [68], belonging [83], ownership [83], and home [68]. In contrast, those who lacked a sense of ownership often felt homeless [62, 69, 80]. Not everyone achieved ownership conventionally. Teenagers in crisis housing asserted ownership through graffiti and vandalism [63], while those sleeping rough claimed their space through domestic practices such as sweeping and decorating their dwellings [47, 62, 68].

#### 4.2.4 Enacting Self‐Determination

People described home as a symbol of independence [59, 69, 71, 72, 75, 77], choice [16, 33, 52, 67, 68, 69, 75, 81, 83], self‐determination [4, 67, 70, 79], autonomy [51, 52, 56, 69, 71, 72, 81, 83], freedom [33, 50, 52, 53, 56, 59, 65, 68, 69, 70, 80, 81, 83] and control [44, 56, 57, 59, 62, 67, 68, 69, 70, 71, 72, 73, 75, 77, 79, 80, 81, 83], over their surroundings [16, 52, 81] and daily lives [52, 55, 67, 68, 69, 70, 71, 75]. Restrictive environments such as homelessness, abusive homes, crisis housing, or types of temporary housing imposed rules and limited self‐determination, thereby limiting the experience of home [4, 46, 55, 56, 63, 66, 67, 68, 69, 70, 71, 75, 76, 77, 83]. Having autonomy over actions [44, 50, 59, 65, 69, 70, 71, 81, 83] and freedom to make choices [4, 46, 55, 59, 66], were crucial ingredients for home [55, 59, 66, 69, 71, 75, 81, 83]. People also expressed a desire to control the timing [52, 59, 65, 66, 68, 69, 70, 71, 81, 83] and location [52] of meaningful activities [16, 67, 83]. These activities included sleep [59, 65, 68, 69, 83], bathing [59, 65, 69], medication [66], dressing in preferred/comfortable clothing [55, 59, 65, 83], cooking and eating preferred foods [52, 69, 75, 83], as well as social and leisure activities like watching tv [59, 69, 70, 71], decorating [16, 81], inviting friends and family over [59, 68, 83], having pets [21], smoking tobacco [59, 65], and use of other drugs without shame or guilt [21].

### 4.3 Home Is Having a Connection

This theme describes how home facilitates connections. This experience encompasses abstract ties to culture and spirituality [44, 45, 50], as well as connections to loved ones and community [52, 79, 80]. Additionally, routines and occupations support the formation of connections to place [16, 47, 51, 54, 67, 69, 80]. The five subthemes illustrate how different facets of home enable people to feel connected.

#### 4.3.1 Connecting to Something More

Home was not always tied to a physical shelter [44, 45, 50, 51, 56, 59, 72] but was based on past experiences [46, 56, 57, 59, 68, 69, 72, 81] and connections to them [16, 33, 50, 52, 55, 56, 58, 59, 63, 68, 69, 80]. Experiences encompassed feelings [46, 82], past relationships [56], occupations [69], and places they had lived [56, 69, 72, 81]. Home can be based on a memory [56, 57], influencing perceptions and expectations [56] on what “true home“ [56]^(p. 152)^ is. Those with traumatic past homes [51, 59] and chronic homelessness [44], described home as an internalised construct [44, 51, 54, 59, 72, 75] through the “bodily sensations of home” [51(p] [73]. including smells, sounds and touch [59, 66, 72]. e.g., one person reflected, “you got to smell bacon…hear laughter…that's home.” [72]^(p.368)^ Some people from Hawaii and North America viewed home through their spiritual and cultural connection to land and waterways [44, 45, 50]. “I'm just houseless…we don't have a shelter but this is home. Hawaii is home.” [50]^(p.78)^

Others described home (and homeless) as a state of mind [44, 56, 64]. This state of mind was sometimes simply a positive emotional environment [56, 58, 59, 60, 62], where they felt happy [56, 59, 81]. One person articulated that “home, basically is the earth,” [44]^(p.765)^ for others, it was the city [44], world [44], or “where you come to God.” [50]^(p.79)^ This expansive view of home was typical of people leading mobile lifestyles [44], who avoided fixed shelter [56]. For these individuals, the experience of transitioning from place to place becomes a core part of their identity [56] meaning that home was not one place [56], but a state of mind that they created and which travelled with them [44]. In some instances, the concept of home was tenuous and elusive because of past experiences, reflecting that “because [I've never had a home], I am not sure what that's supposed to be like. It's kind of like if you don't know what love is because you've never felt it” [46] ^(p. 1094)^.

#### 4.3.2 Finding Your People

During homelessness, some people felt like they did not belong [16, 49, 77], feeling alone even when surrounded by others [60, 62]. This detachment [59, 60, 62] was so profound that one person remarked, “it's like you are dead.” [59]^(p.140)^ Positive [16, 56, 67, 81], warm [58] relationships with children [4, 53, 54, 55, 56, 59, 64, 80], biological families [53, 56, 64, 80], surrogate families [44, 46, 49, 52, 56, 58, 59, 66], partners [44, 81], friends [4, 44, 46, 53, 56, 59, 81], neighbours/other residents [46], staff [21, 46, 52, 54, 56, 59, 66, 67, 81], workmates [58], volunteers [50] and pets [54, 67, 73, 81] helped to transform unlikely places [21, 81] like crisis housing into home [16, 21, 46, 50, 53, 55, 56, 58, 59, 63, 64, 67, 68, 80, 81]. Queer families became significant for LGBTQIA+ people whose homelessness often resulted from being disowned by biological families [74]. Restrictions in crisis housing often interrupted the restoration of relationships by deterring visitors [55, 71, 73], leading to loneliness [71], loss of community [46], and discrimination [69]. For women who had experienced violence in their former living situations, moving/getting to know a new area and establishing new relationships, or going back to a former area to be close to family or friends supported a sense of belonging [4], but for some leaving an ‘imperfect’ home could also mean leaving behind a place that was familiar and they were connected to [21]. During and after homelessness, socially excluded people often found belonging within subculture communities [44, 65, 74].

#### 4.3.3 Feeling Loved

Feeling at home was reinforced through mutually given [44, 56, 58, 60, 80, 81] love [44, 49, 56, 58, 59, 60, 80, 81], support [33, 52, 56, 58, 59, 67, 71, 81], care [49, 50, 56, 59, 60, 61, 62, 80, 81], and comfort [33, 58]. People re‐established home through reconnecting with family structures [56]. Some felt so familiar with staff that they described them as like a family member such as “*another mum*” [56]^(p.171)^ or sister [66] and felt adopted by them [53], while also taking on new roles for themselves such as a mother [56] or “*older sister*.” [58]^(p.32)^ Some people defined home through their relationships with children [53, 55, 80, 81], parents [81], spouse [44, 81], or pets [62, 67, 73, 81]. Some who did not experience home equated it with a lack of such relationships [51, 68]. For others, neglectful and abusive relationships caused them to become homeless while still living in their former homes [4, 21, 44, 56, 59, 60].

#### 4.3.4 Experiencing Community Connection

Home was an inclusive [71] place where people belonged [16, 44, 49, 52, 54, 56, 71, 74, 80], felt accepted [44, 49, 54, 56, 77], and connected to a community [16, 52, 54, 66, 71, 80]. This connection was often achieved through social participation [44, 47, 52, 53, 54, 63, 66, 67, 70, 71] that contributed to home [16, 52, 56, 71, 77, 80]. Community participation mitigated feelings of social marginalisation [49] and created normalcy [54, 70, 71]. This was the same for people who were experiencing homelessness and created ‘good‐enough homes in un‐homelike places’ [21] by having a group of people who cared for each other and were socially connected [21]. Due to histories of discrimination [49, 52, 55, 74], stigmatisation [49, 55, 67] and rejection from more normative communities [49] homeless people often felt socially excluded [49, 52, 62, 67]. When exiting crisis housing [66, 68, 71] important connections formed there were often lost [49, 66, 68], and establishing new ones in unfamiliar neighbourhoods proved challenging [71]. This disconnect from both people and place [16, 66, 69] could result in people never leaving [49] or returning to homelessness and crisis housing [16, 49] where they felt more at home due to the social connections they had there [21, 69]. In contrast, others developed strategies to enhance social engagement upon arriving in new neighbourhoods [16, 62], such as using public transport, and connecting with neighbours via gift giving [16]. One individual even grew an impressive garden which attracted tourists to their home [16].

#### 4.3.5 Engaging in Routine and Occupations

Having a stable dwelling facilitated routines [16, 47, 51, 52, 56, 67, 69, 70, 73, 76, 81] but was not always a necessity [47, 51]. Routines aided social [4, 16, 80] and community connection [4, 16, 47, 48, 51, 54, 67, 80], purpose [16, 21, 67, 82], and belonging [4, 16, 69, 80] thus rebuilding identity [16, 21, 56, 73], independence [82], sustaining tenancy [16], supporting sobriety efforts [16] and maintaining a positive mood [56]. Routines were sometimes driven by a desire for completing occupations [21] and solitude [67, 69, 80, 81]. Maintaining a routine could be challenging due to the discipline required [16, 44]. Having structured routines [16] such as employment [51], caring for a pet [16, 21], or simply relaxing [16] kept people meaningfully occupied. Routines and rituals provided community connection including taking the bins out for their neighbours and preparations for festivities [21].

Having a home meant participating in occupations which are outlined in Table 8. Completing homemaking occupations fostered positive self‐respect [44, 51, 66, 81, 83] and perceptions of normalcy [21, 48, 69, 77] which instilled pride [77, 83]. Being in crisis housing where domestic chores, for example shopping for laundry supplies were done for them, undermined the opportunity for esteem and competency [63]. Many people took on new occupations within their community [59], driven by a desire to contribute [67], connect [21], and find purpose [16, 54, 67, 70]. Engaging in social participation and shared occupations built social connections [16, 52, 54, 67, 71]. Barriers to occupational participation included materials/resources being insufficient to create home [48], financial constraint [16, 67] and disturbances/rules within crisis housing [4, 56, 63].

**Table 8:** Occupations that supported home.

Category of occupation	Examples of occupations
**Leisure**	Watching TV [16, 56, 63, 67, 69, 81, 83], relaxing, [16, 69, 75, 81]listening to music [63, 82, 83], looking at a phone [16, 63], reading [83], drawing [82], art [82] and gardening [48]
**Social participation**	Socialising with housemates, guests [16, 33, 54, 63, 67, 69, 75], crisis housing residents, friends, family, neighbours [16, 52, 54, 56, 68]. Eating [44, 47, 53, 66, 70], smoking [52, 63, 66] playing cards [52] with others. Participating in music groups [79] and recreational activities in parks [16, 21, 56, 70]
**Religious and cultural participation**	Attending church [52, 80], culturally specific occupations [76, 80], living off the land [45, 80]
**Activities of daily living**	Bathing [59, 69, 70, 80, 81], meditation [71], preparing and eating food [16, 33, 52, 54, 66, 75]
**Instrumental activities of daily living**	Housework [16, 63]; cleaning [16, 53, 83], laundry [63, 69, 70], making the bed [16], grocery shopping [16, 63, 70], looking after animals [21, 82]
**Work/Education**	Volunteering [16, 54, 56, 59, 67, 70], working [16, 56, 59, 67, 70, 80], studying [16, 56, 67], collecting cans for recycling [21]

## 5 Discussion

The meta‐synthesis included 44 studies and explored the perspectives of more than 1023 people who have experienced homelessness, focusing on how they describe the concept of home. From these descriptions, there were three themes constructed that explained what was desired and gained when attaining home for the participants, and ways in which personal circumstances shape and enable the ability to establish a home. These themes were ‘home is stability and return to self’; ‘home is having a protective and controllable threshold’ and ‘home is having connection.’

Theme one explained the role of a consistent *stable foundation* for experiencing home and a subsequent ‘return to self’. A meta‐aggregation of qualitative research reveals how home influences self‐discovery [2]. Padgett [70] discusses how ontological security, achieved through a stable base, provides the conditions for *identity* to be reconstructed. Past experiences of instability can shape expectations of continued uncertainty which then perpetuates future instability [84] leading to “psychological homelessness” [85] ^p. 185^ even if housed. Although youth under 16 years were not included in this review, this expected future instability based on past experience aligns with literature exploring experiences of homeless youth [84]. To establish stability, there is a need for trusting relationships with any health or social care professionals and the homeless person they are helping to house. This approach is reflected in the Bridging the Transition to Housing framework developed by Marshall et al. [10]

Being able to establish a stable home requires a process of self‐reflection and the ability to repair previous social roles or create new ones [70]. Arguably, carving out new roles enables people to reconnect with themselves, thereby stabilising their *identity*. Through reflection, people can gain deeper insight into their past and future, which improves their current self‐perception [2]. While stability is essential to feeling at home and discovering identity, stability requires more than shelter and is also determined by feeling psychologically and emotionally *safe and protected*, with *connections* to place. Seager [86] argues for prioritising psychological needs in support services for homeless people, emphasising that a psychological‐based care system is essential to developing secure emotional attachments. For some, addressing this attachment and need for connection (emotionally and relationally) must come before being ready to establish a stable place to live (their future home).

Theme two described home as a boundary that provides *protection (physical and emotional)* from the outside world. To regain the ability to experience home, people who have been homeless need to feel protected through establishing boundaries. Participants enjoyed being in their own space away from surveillance and were able to control who entered their homes. This finding was highlighted by Henze‐Pederson et al., [4] who found that previously homeless women escaping abusive situations often created protective boundaries in their new homes, beginning with small actions such as buying curtains. Homes were described as sanctuaries for healing and recovery. Many expressed that this boundary enabled them to engage in self‐care and therapeutic practices, such as meditation and self‐reflection.

While Marshall et al. [87] did not directly focus on the description of home, they highlighted that individuals began processing trauma once in stable housing [87]. Simply having a stable, protected emotional space, allowed participants to engage in emotional processing, something that had been impossible while unhoused. While there is a small amount of literature [2, 16, 88] exploring these therapeutic self‐care practices, it does not elaborate on how these practices are carried out. It seems clear, however, that providing stable housing alone is insufficient and there is a necessity to provide supports and conditions for feeling safe and protected, so *routine occupations* and establishment of positive social connections can occur. This observation is supported by McCarthy [88] who saw direct connections between the social milieu of a homeless person and their identity; meaning that people may experience identity change once exposed to different social connections in new housing.

Theme three described the importance of connectedness such as relationships, *inclusion*, and belonging with others. Many people experiencing homelessness built support networks and connections in crisis housing [65, 66]. However, once they left these environments, these connections were often severed [16, 49, 68] leading to feeling more socially isolated than they did before having the temporary housing [87, 89]. A meta‐aggregation described the challenge of social isolation as particularly significant during the initial period of receiving housing [89].

Once the recently housed person establishes feelings of safety, they can begin reaching out for *social connections* [89] and engaging in shared meaningful *occupations* [89]. Nichols [90] found that the experience of being housed was challenging for some people due to adjusting to a new pace of life and the “unbearable quiet” [2] ^p. 15^. Similarly, Aubrey [85] saw limited mental health benefits in the provision of supportive housing for homeless people in high‐income countries. The present study suggests that engagement in meaningful occupations and social connectedness may be considered essential determinants to counteracting any negative effects after being housed.

## 6 Strengths and Limitations

This systematic review is the first to undertake a synthesis of research bringing together findings from qualitative research about the meaning of home for people who have or are homeless. A strength of the review is the systematic search of seven databases and grey literature. Screening and critical appraisal were conducted in duplicate and reporting guides were used to reduce bias. The first, second and third order analysis was done with rigour using robust methods to reduce the data to a series of themes without losing richness or contextual details. A check of the themes was implemented by a consultant expert in the area, who gave valuable feedback on the language and structure. To minimise bias in interpretation, the research team engaged in regular reflexive discussions during the review process, particularly during data analysis. Due to the high number of included studies, performing reference list checking for further inclusions was not conducted, meaning some papers may have been missed. Due to the focus of the review, research that did not explicitly focus on the meaning of home (n = 56) or did not describe home (n = 16) were excluded even though they may have had some data to contribute. Most participants in the included studies had access to supports and housing when interviewed, resulting in data underrepresenting the perspectives of those who remained homeless.

The synthesis brought together the experiences across different ages (between 16 and 65), genders and cultural backgrounds. Achieving variation in the sample is a strategy and strength of qualitative research as it brings heterogeneity into the findings; however, this approach means distinctions in experience for different sub‐sections of the population are not reported. For example, the experiences of women escaping domestic violence were not reviewed separately from the experiences of homeless men. Only papers published in English were included which meant that most participants were from English speaking countries. This methodological decision meant that experiences reflected in the data are from the global North (apart from one study from the Philippines). Experiences of people living in non‐English speaking countries, including the global South are not captured in this synthesis. This research also excluded studies (n = 10) about cultural homelessness including internally and internationally displaced refugees and migrants. These omissions may limit the generalisability of the findings to broader cultural contexts, and it is recommended that future research explore these gaps.

## 7 Recommendations for Policy, Practice and Future Research

**Figure 2:**
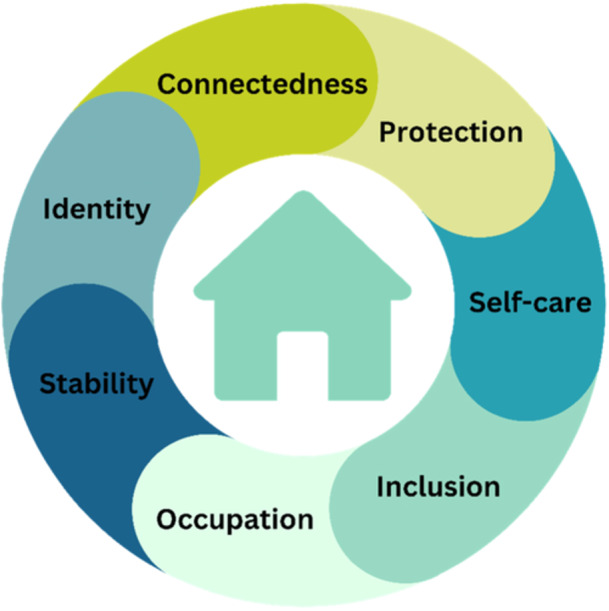
Key determinants for homeless people to feel at home.

This review provides an insightful contribution to the literature regarding the means, supports, or strategies that can effectively facilitate social connections and occupational engagement for recently housed homeless people. There appear to be seven key concepts that arise from these themes which summarise the contextual and nuanced meaning of home for people who have been or are homeless. These concepts are stability, identity, connectedness, protection, self‐care, inclusion, and participation in occupations (see Figure 2). Existing transition to housing programmes may focus on the physical housing needs such as furniture and food, but these more psychosocially oriented concepts provide a framework for considering how to provide targeted services and support for a person to successfully transition into housing and avoid returning to homelessness. Based on this meta‐synthesis these seven concepts are found to be inter‐connected requirements and key determinants for this population to thrive and feel at home in housing. Wherever newly housed formerly homeless people struggle settling into their new environment, it is possible that one of these key determinants may be lacking. These concepts could be addressed in policy development and lead to a series of recommendations for practice and research aimed at improving the transition to housing and helping people who are, or have been homeless, feel at home in their houses. Without this research, unsuccessful housing transitions may be unfairly attributed to the individuals, with inadequate attention to determinants in policy and practice. These concepts also provide a firm basis for further research including exploration of how these concepts may interrelate and be prioritised differently depending on age, gender, cultural background and differing past experiences.

### 7.1 Self‐care

The newly housed person may be unaccustomed to providing self‐care, but it seems provision of opportunities for self‐reflection builds identity. Support services could prompt and provide strategies for different ways of learning more about personal identity, values, preferred occupations and who they would like to connect with. Further research is needed to understand the value of solitary reflection and self‐care practices and the barriers and enablers to these practices for people entering housing.

### 7.2 Identity

Engagement in new occupations associated with being housed may influence the (re)discovery of identity and support feelings of inclusion when housed. Further research is needed to explore the links between identity (re)formation and being ‘at home’.

### 7.3 Occupation

It is recommended that policymakers and practitioners consider the role of performing occupations to support psychological stability, potentially in anticipation of securing housing and planning for what people will do once they are housed. This will require investment in allied health, such as occupational therapy and social work to provide this support to homeless people before, during and after transition into housing.

### 7.4 Stability and Protection

For housing providers/authorities and policy makers to explore what home means for the person they are housing, they need to work toward supporting feelings of stability, safety and protection in housing. If the person being offered housing, declines what is being offered, this feedback could inform improved future housing selections, ensuring options align with needs to maximise the chance of a successful transition.

### 7.5 Connectedness

Social connectedness and belonging seems pivotal for thriving in housing and feeling at home. This connectedness may not always happen naturally and may need facilitation and support from community organisations and formal service providers until the connections and constructive community participation and integration is established. Further research is needed to enhance knowledge of successful methods and strategies for creating social connections and community participation.

## 8 Conclusion

Many people who have been homeless find the transition into housing challenging meaning they may return to homelessness. To provide insights into why this may occur this research explored how people who have experienced homelessness between the ages of 16 – 65 describe the concept of home. These insights may improve policy, and practice, and provide a framework for further research. The review synthesised the findings from 1023 people in 44 studies undertaken internationally. The research presented three themes that described the concept of home for people who have been homeless as more than a passive shelter to be inhabited but a place that is made, within which to dream, feel safe, healthy, happy, and be a part of society. Home is a foundation that allows thriving through self‐care, stability, protection, connectedness, identity development, engagement in occupation and inclusion. These concepts provide determinants for supporting people who have been homeless to feel at home in housing. It is recommended that the inter‐relatedness of these concepts be explored further with different populations, cultures and background experiences, and that programmes supporting homeless people transitioning into housing address these psychosocial concepts in practice.

## Author Contributions

**Leila Thornhill:** conceptualisation, data curation, formal analysis, investigation, methodology, visualisation, writing – original draft, writing – review and editing, project administration. **Ben Sellar:** conceptualisation, methodology, data curation, investigation, project administration, supervision, formal analysis, writing – review and editing, visualisation. **Gabrielle Rosa Hernandez:** conceptualisation, data curation, formal analysis, investigation, visualisation, writing – review and editing, supervision, project administration, methodology. **Carolyn M Murray:** conceptualisation, data curation, formal analysis, investigation, methodology, project administration, supervision; visualisation; writing – review and editing.

## Funding

The authors have nothing to report.

## Ethics Statement

The authors have nothing to report.

## Consent

The authors have nothing to report.

## Conflicts of Interest

The authors declare no conflicts of interes.

## AI Statement

Generative AI has not been used in the preparation of this article.

## Supporting information

Supporting File

## Data Availability

Data available on request from the authors.
