# Supplementary material for: ‘Home Is Where You Are Together’: Qualitative Systematic Review and Meta‐Synthesis of Homeless People's Description of Home
Source: Health Expect. 2026 May 5;29(3):e70683. doi: 10.1111/hex.70683 (PMC13142092; doi:10.1111/hex.70683)
Supplement: Supplementary file 1 — Supporting File [file HEX-29-e70683-s001.docx]

Supplementary file 1: Medline search strategy

| **Database** | **Search terms** | **Limits applied** | **Results** |
| --- | --- | --- | --- |
| **Medline** | S1 - Ill-Housed Persons/ S2 - Homeless Youth/ S3 - (homeless* or houseless or unhouse* or unsheltered or undomiciled or roofless* or rootless* or rough sleeper* or rough sleeping or street entrenched or street involved or squatt* or shelterless or drop in center* or drop in centre* or derelict* or vagabond* or gypsy* or nomad* or itinerant* or drifter* or bag person*).ti,ab,kf. S4 - ((unstable or unhealthy or unsafe or inadequate or improvised or insecure or instability or emergency or temporary or overcrowded or supportive) adj3 (accommodation* or hous* or dwelling* or shelter*)).ti,ab,kf. S5 - ((street or transient or homeless* or internally displaced or ill housed or roofless* or rootless* or houseless*) adj3 (person or persons or people or youth* or family or families or woman or women or man or men or child* or teenager* or adolescen* or adult*)).ti,ab,kf. S6 - 1 or 2 or 3 or 4 or 5 S7 - (home or home making or homemaking or homely or homey or homier or homiest or homeyness or hominess or feel* homely or felt homely or real home or ideal home or home like or home comfort or belonging or connection).ti,ab,kf. S8 - ((idea or ideal or feeling or meaning* or sense or feel* or felt or experience or place or place to call or felt or like or is where or call it or make* or description or definition or exploration or perception or notion or emotion or significance or concept) adj1 (of home or at home or like home or of home or is home or a home or home)).ti,ab,kf. S9 - 7 or 8 S10 - Grounded Theory/ S11 - Qualitative Research/ S12 - Interview/ S13 - Personal Narrative/ S14 - Narration/ S15 - Focus Groups/ S16 - Interviews as Topic/ S17 - "Surveys and Questionnaires"/ S18 - (qualitative stud* or qualitative research or qualitative analysis or qualitative or grounded theory or narrative* or narrative analysis or narrative approach or narrative inquiry or naturalistic inquiry or thematic analysis or discourse analysis or comparative analysis or ethnograph* or case stud* or community based research or community-based research or focus group* or interview* or participant observation* or photographic diar* or photovoice or phenomenolog* or participatory action research or participatory research or action research or mental recall or survey* or questionaries or face to face interview* or face-to face interview*).ti,ab,kf. S19 - 10 or 11 or 12 or 13 or 14 or 15 or 16 or 17 or 18 S20 - Attitude/ S21 - Observation/ S22 - Thinking/ S23 - Emotions/ S24 - (view* or viewpoint* or point of view or experience* or expectation* or express* or encounter* or account* or attitude* or belief* or believes or reflection* or feel* or understand* or perception* or perspective* or perceive or opinion* or observation* or outlook* or insight* or world view or worldview).ti,ab,kf. S25 - ((personal or life or lived) adj1 (history or story or stories or narrative* or experience*)).ti,ab,kf. S26 - 20 or 21 or 22 or 23 or 24 or 25 S27 - 6 and 9 and 19 and 26 S28 - limit 27 to english language | Language: English | 569 |
